# Use of Intravenous Clevidipine in the Management of Severe Preeclampsia: A Case Report

**DOI:** 10.7759/cureus.99287

**Published:** 2025-12-15

**Authors:** Cody Brazeal, Anne Shapiro

**Affiliations:** 1 Anesthesiology, Kaweah Health Medical Center, Visalia, USA

**Keywords:** case report, clevidipine, hypertensive emergency, preeclampsia, pregnancy

## Abstract

Severe preeclampsia is a hypertensive disorder of pregnancy that poses significant risks to both maternal and fetal health. First-line antihypertensives such as labetalol, hydralazine, and nifedipine are widely used; however, alternative agents may be considered in refractory cases. Clevidipine, an ultrashort-acting intravenous dihydropyridine calcium channel blocker, offers rapid titratability and is used in critical care settings, although its use in pregnancy remains understudied. We present a case of successful clevidipine use in a patient with severe preeclampsia refractory to standard therapy, highlighting its potential role in obstetric hypertensive emergencies. Clevidipine was selected due to its rapid onset, short half-life, and capacity for precise, titratable blood pressure control. Additionally, its metabolism by blood and tissue esterases, independent of hepatic or renal function, makes clevidipine advantageous in preeclampsia, where hepatic and renal dysfunction are often part of the disease process. Its rapid titratability also allows prompt discontinuation following spinal anesthesia, preventing prolonged hypotension that may result from non-titratable agents.

## Introduction

Preeclampsia complicates approximately 5-8% of pregnancies and remains a leading cause of maternal and perinatal morbidity and mortality worldwide [1]. Severe preeclampsia is defined by systolic blood pressure ≥160 mmHg or diastolic blood pressure ≥110 mmHg, accompanied by evidence of end-organ dysfunction [2]. Uncontrolled severe hypertension carries substantial maternal risk, including stroke, pulmonary edema, placental abruption, and multiorgan dysfunction, while also threatening fetal well-being through uteroplacental insufficiency, growth restriction, and potential fetal demise.

The American College of Obstetricians and Gynecologists (ACOG) recommends intravenous labetalol, intravenous hydralazine, or oral immediate-release nifedipine as first-line therapy for acute severe hypertension in pregnancy [2]. Refractory hypertension in preeclampsia may arise from severe endothelial dysfunction, heightened vasoconstrictor activity, intravascular volume shifts, or altered pharmacologic responsiveness, leading some patients to remain hypertensive despite guideline-recommended agents. In situations where these agents are ineffective or contraindicated, alternative agents may be considered.

Clevidipine butyrate (Cleviprex) is an ultrashort-acting intravenous calcium channel blocker that reduces blood pressure by inhibiting L-type calcium channels in vascular smooth muscle, producing arterial vasodilation and lowering systemic vascular resistance. It is rapidly metabolized by esterases, resulting in a half-life of one to two minutes [3]. A review of the existing literature revealed no prior reports describing the use of clevidipine in pregnant patients with preeclampsia. This absence of data underscores the novelty of the present case and highlights the potential utility of clevidipine in obstetric hypertensive emergencies when standard agents are ineffective.

## Case presentation

A 29-year-old G1P0 woman with a past medical history of uncontrolled hypertension and obesity presented at 29 weeks and two days of gestation to labor and delivery triage after referral by her maternal-fetal medicine specialist for evaluation of severe preeclampsia. Her blood pressures were persistently elevated, with a presenting blood pressure of 224 / 130, despite adherence to oral labetalol 300 mg three times daily (Table 1). She denied headache, visual changes, chest pain, or focal neurologic symptoms. Fetal monitoring demonstrated appropriate variability without decelerations. The patient was alert, oriented, and in no acute distress. She had an elevated BMI of 48.8 kg/m² (125 kg, 160 cm). Airway assessment revealed a Mallampati class III. The head was normocephalic and atraumatic. Respirations were non-labored with symmetric chest wall expansion, and cardiovascular examination showed a normal rate and regular rhythm. The abdomen was gravid, and there was no peripheral edema.

**Table 1:** Perioperative Blood Pressure and Intervention Timeline PACU, post-anesthesia care unit

Time	Blood Pressure (mmHg)	Event / Intervention / Notes
11:14	224 / 130	Initial presentation
11:18	223 / 135	
11:30	208 / 108	Hydralazine 10 mg IV given
11:41	182 / 105	
11:42	-	Clevidipine infusion started at 0.5 mg/hr
11:46	166 / 99	
11:51	153 / 90	
11:56	150 / 88	
12:01	146 / 81	Clevidipine decreased to 0.4 mg/hr
12:06	145 / 80	
12:11	148 / 86	
12:16	137 / 79	Clevidipine decreased to 0.1 mg/hr
12:23	146 / 90	
12:26	141 / 102	
12:27	-	Spinal anesthesia administered
12:30	116 / 69	
12:31	-	Clevidipine infusion discontinued
12:48	101 / 57	
12:57	-	Female neonate delivered
13:03	100 / 59	
13:15	120 / 65	
13:28	124 / 68	
13:36	129 / 93	Arrived at PACU

One week earlier, the patient had been admitted for elevated blood pressure that initially responded to standard therapy. She received antenatal corticosteroids for fetal lung maturation and was discharged on oral labetalol 300 mg three times daily.

On the day of presentation, her blood pressure remained severely elevated despite maximal oral therapy. Laboratory values were notable for a platelet count of 228 × 10⁹/L, creatinine 0.75 mg/dL, aspartate aminotransferase (AST) 62 U/L, alanine aminotransferase (ALT) 32 U/L, and alkaline phosphatase 95 U/L. Additionally, a urine creatinine of 73.8 mg/dL and urine protein of 25.5 mg/dL yielded a urine protein-to-creatinine ratio of 0.35.

Differential diagnosis considerations included gestational hypertension, hemolysis, elevated liver enzymes, and low platelet count (HELLP) syndrome, and secondary causes such as pheochromocytoma or glomerulonephritis; however, the combination of new-onset severe hypertension after 20 weeks, mild transaminitis, and proteinuria confirmed the diagnosis of severe preeclampsia.

The patient was given 10 mg of intravenous hydralazine and started on intravenous magnesium sulfate. In light of the imminent risk of maternal complications, the decision was made to proceed with emergent cesarean delivery. Given the need for immediate blood pressure control, the patient’s inadequate response to both oral labetalol and intravenous hydralazine, and the preference for an agent independent of hepatic or renal metabolism, clevidipine was initiated. Additionally, use of a titratable agent allowed controlled blood pressure reduction before neuraxial anesthesia and permitted rapid down-titration afterward, helping prevent prolonged hypotension that can occur with longer-acting medications such as hydralazine.

Clevidipine infusion was initiated at 0.5 mg/hr, resulting in prompt and controlled blood pressure reduction, with continuous reassuring fetal heart rate monitoring. After 19 minutes, the infusion was decreased to 0.4 mg/hr. The rate was then titrated down to 0.1 mg/hr in anticipation that the spinal anesthetic would further lower blood pressure. Shortly after the spinal anesthetic was placed, the clevidipine infusion was discontinued, having run for a total of 49 minutes, with a total dose of 0.29 mg administered. The patient remained normotensive for the remainder of the case.

Of note, the female neonate, weighing 1,075 g, had APGAR (Appearance, Pulse, Grimace, Activity, and Respiration) scores of six and seven at one and five minutes, respectively, and was admitted to the neonatal intensive care unit for prematurity-related care. The neonate was intubated and gradually weaned from mechanical ventilation to noninvasive support, progressing to room air before discharge on day of life 62, at a corrected gestational age of 37 and 1/7 weeks.

Postoperatively, the patient remained normotensive in the post-anesthesia care unit (PACU) without evidence of rebound hypertension. Over postoperative days one to five, she again became hypertensive, prompting stepwise adjustments to her antihypertensive regimen. By postoperative day five, she was maintained on oral labetalol 200 mg and nifedipine XL 60 mg daily, with a maximum recorded blood pressure of 135 / 98 mmHg on the day of discharge.

## Discussion

This case demonstrates the potential role of clevidipine in the management of obstetric hypertensive emergencies when standard therapy proves inadequate. ACOG guidelines designate labetalol, hydralazine, and nifedipine as first-line therapies [2]; however, refractory cases highlight the potential need for alternative agents.

Pharmacologic advantages of clevidipine

Clevidipine offers pharmacologic advantages, including a rapid onset of action within two to four minutes, an elimination half-life of approximately one minute, and minimal accumulation with prolonged infusion [3]. These properties allow for precise titration and rapid discontinuation, making it attractive for scenarios in which blood pressure must be tightly controlled. In perioperative and neurocritical care settings, clevidipine has demonstrated superior speed and precision of blood pressure control compared with alternative agents [4].

Additionally, compared with standard therapies, clevidipine offers pharmacokinetic advantages through its rapid, organ-independent clearance. Labetalol and nifedipine are primarily cleared hepatically via glucuronidation and CYP3A4 oxidation, respectively [5], with increased metabolic clearance during pregnancy due to enzyme induction and elevated hepatic blood flow. Hydralazine is also hepatically metabolized by acetylation [6], with limited renal contribution. In contrast, methyldopa undergoes both hepatic metabolism and renal excretion [7]. These differences underscore clevidipine’s unique profile of organ-independent clearance, as it is rapidly hydrolyzed by blood and tissue esterases rather than hepatic or renal pathways, providing more predictable kinetics in patients with potential end-organ dysfunction.

Safety profile

Nevertheless, its use in pregnancy is limited, and safety data remain sparse. According to current FDA labeling for clevidipine, human data are insufficient to determine drug-associated risks for adverse maternal or fetal outcomes, although poorly controlled hypertension itself is known to increase risks of maternal stroke, fetal growth restriction, and death. In animal studies, clevidipine caused fetal deaths and skeletal delays at high doses but no structural malformations [3]. These studies were conducted using doses 2.8-7.6 times higher than expected human exposure, far exceeding the dosage administered in this case. Nonetheless, use during pregnancy is advised only when the potential maternal benefit outweighs potential fetal risk.

Clevidipine was previously classified as pregnancy category C. Since 2015, the FDA’s Pregnancy and Lactation Labeling Rule has replaced the former letter-category system with narrative summaries addressing pregnancy, lactation, and reproductive potential, promoting a more individualized assessment of risks and benefits [8]. Of note, under the prior system, many standard antihypertensive agents were also designated as category C, not due to proven harm, but because adequate human data were lacking. Their well-documented safety and efficacy over decades underscore that the former pregnancy category labels offered limited interpretive value compared with accumulated clinical data.

Adverse effects of clevidipine include reflex tachycardia and hypertriglyceridemia with prolonged infusions, and its lipid emulsion formulation contraindicates use in patients with egg or soy allergy [3]. Compared with established agents such as labetalol, hydralazine, methyldopa, and nifedipine, clevidipine’s pregnancy safety profile in humans remains theoretical. Its rapid onset, ultrashort half-life, and organ-independent metabolism make it a possible option in refractory cases, but its use remains cautious and highly selective given the limited clinical evidence.

Previous reports of clevidipine use in pregnancy and postpartum

One prior report has documented the successful use of clevidipine in a postpartum patient with preeclampsia and subarachnoid hemorrhage [9]. To our knowledge, the present case constitutes the first published description of clevidipine administration in a pregnant patient. Considered together, these cases underscore the potential applicability of clevidipine in carefully selected obstetric patients requiring rapid and titratable blood pressure control.

Theoretical implications for uteroplacental blood flow

Although data regarding clevidipine in pregnancy are lacking (no controlled studies to date in parturients), the known physiology of uteroplacental perfusion suggests that any intervention that significantly lowers maternal systemic vascular resistance may have dual effects: by reducing afterload and improving maternal cardiac output, it may enhance uterine perfusion; however, if maternal blood pressure falls excessively, the uteroplacental gradient may diminish and fetal oxygen delivery could be compromised [10]. In light of the absence of specific obstetric outcomes data, this theoretical framework suggests that close monitoring may be warranted when using potent arterial vasodilators in pregnancy.

Limitations and future directions

The single-patient design of this report limits the ability to draw conclusions about safety or efficacy in a broader population. Future investigations may incorporate parameters such as maternal blood pressure trends, umbilical artery Doppler velocimetry, fetal heart rate variability, and noninvasive maternal cardiac output monitoring, thereby contributing to a more comprehensive characterization of maternal-fetal safety.

Patient perspective

The patient expressed gratitude for the rapid stabilization achieved before delivery and was satisfied with her postpartum recovery.

Informed consent

Informed consent was obtained for publication of this case report. Efforts were made to remove all patient identifiers in compliance with institutional and journal policies.

## Conclusions

Clevidipine is not included in standard obstetric treatment protocols for severe hypertension but may provide effective, rapidly titratable blood pressure control in cases refractory to conventional therapy. This case highlights several key learning points: clevidipine’s organ-independent metabolism offers predictable kinetics even in the setting of potential hepatic or renal dysfunction; its ultrashort half-life allows precise hemodynamic control and timely discontinuation around neuraxial anesthesia; and its potent arterial vasodilatory effect, while beneficial for rapid afterload reduction, warrants close monitoring to avoid excessive maternal hypotension that could affect uteroplacental perfusion. As the first intrapartum description of clevidipine use, this report adds to the limited literature and underscores the need for further investigation into its maternal and fetal safety in pregnancy.
